# Supplementary material for: Asymmetric Synthesis of Quaternary Hydantoins via a Palladium-Catalyzed Aza-Heck Cyclization
Source: J Am Chem Soc. 2025 Nov 14;147(49):44692–8. doi: 10.1021/jacs.5c16022 (PMC12703750; doi:10.1021/jacs.5c16022)
Supplement: Supplementary file 2 [file ja5c16022_si_002.zip › All NMR FID Files/12/12_AllNMR/1/pdata/1/email_EM04-024-1-F29-end_1_1.pdf]

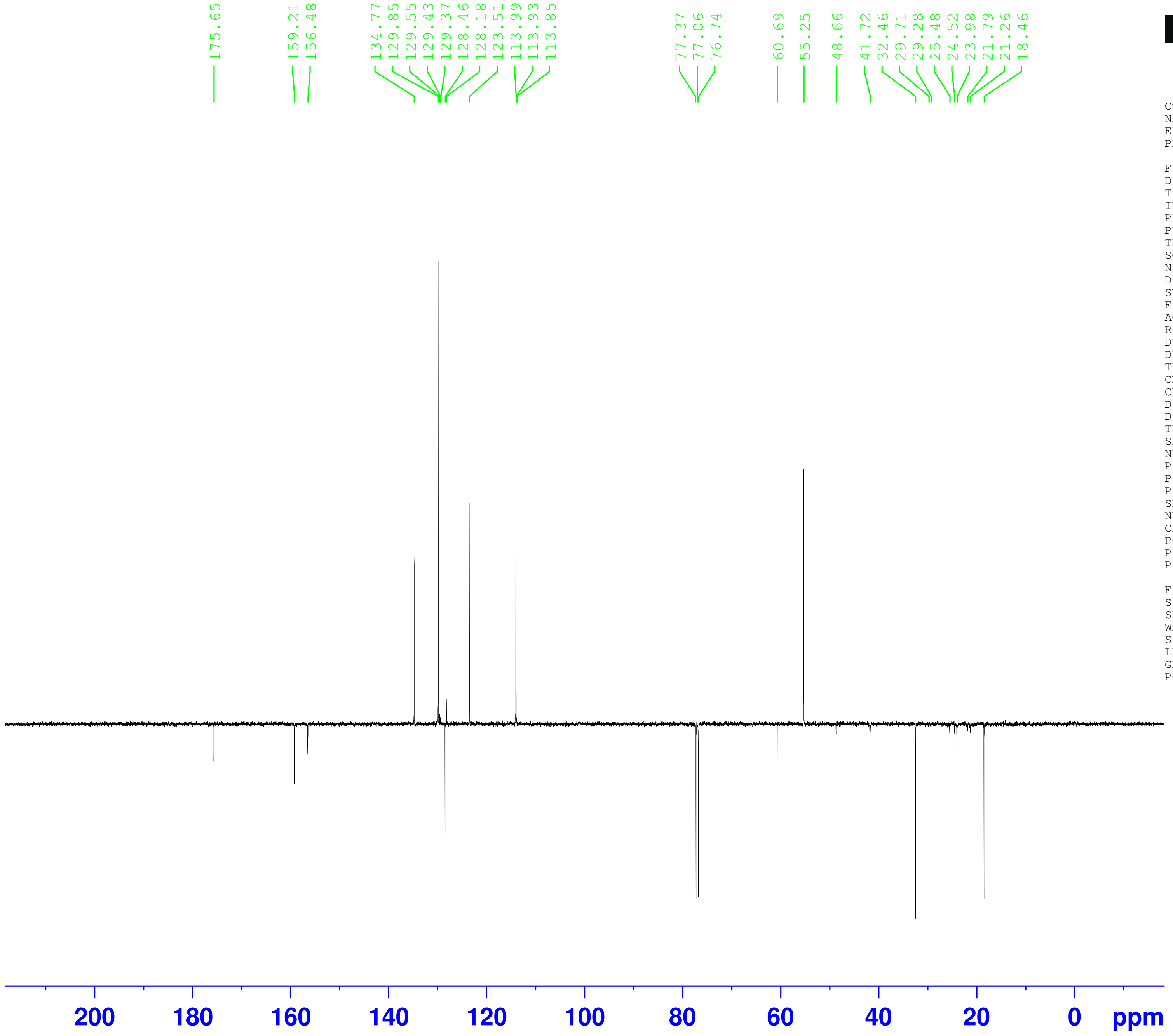

Current Data Parameters
NAME EM04-024-1-F29-end
EXPNO 1
PROCNO 1

F2 - Acquisition Parameters

Date_	20230402
Time	16.30 h
INSTRUM	Avance Neo 400 U of DE Brown Lab
PROBHD	Z824801_0171 (
PULPROG	jmod
TD	65536
SOLVENT	CDCl3
NS	1024
DS	4
SWH	23809.524 Hz
FIDRES	0.726609 Hz
AQ	1.3762560 sec
RG	101
DW	21.000 usec
DE	6.50 usec
TE	298.2 K
CNST2	145.0000000
CNST11	1.0000000
D1	5.00000000 sec
D20	0.00689655 sec
TD0	1
SFO1	100.6228298 MHz
NUC1	13C
P1	10.00 usec
P2	20.00 usec
PLW1	44.54999924 W
SFO2	400.1316005 MHz
NUC2	1H
CPDPRG[2	waltz65
PCPD2	90.00 usec
PLW2	16.44099998 W
PLW12	0.20298000 W

F2 - Processing parameters

SI	32768
SF	100.6127685 MHz
WDW	EM
SSB	0
LB	1.00 Hz
GB	0
PC	1.40
